# Derek Bolton

**DOI:** 10.1192/bjb.2020.98

**Published:** 2021-04

**Authors:** Abdi Sanati

**Figure:**
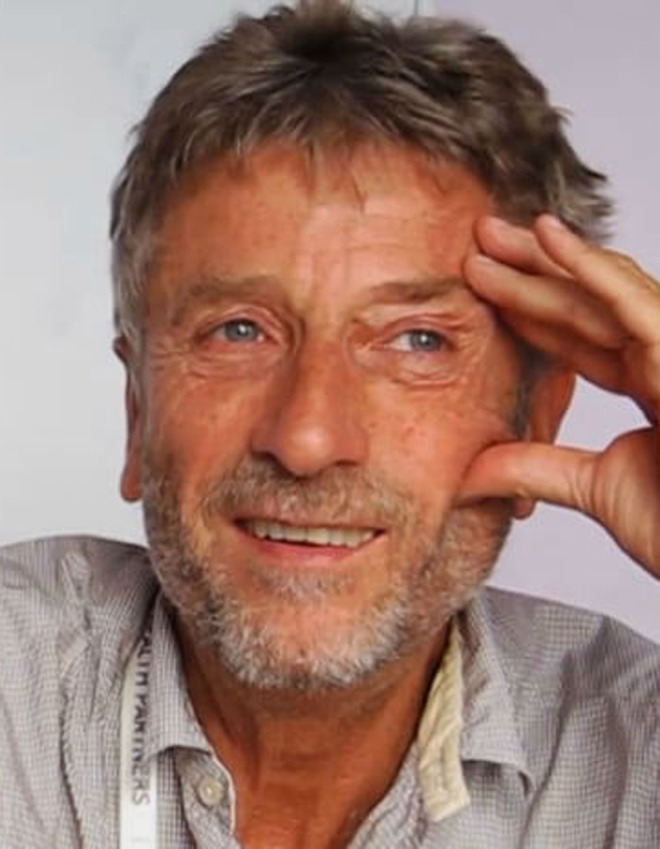

Derek Bolton is Professor of Philosophy and Psychopathology at King's College London, UK. He is one of the pioneers of the discipline of philosophy of psychiatry. He founded the MSc programme in Philosophy of Mental Disorders at King's, which was one of the few in the world. It was while taking that course that I first met him. He has remained a great teacher and mentor for me and many other colleagues throughout the years. His publications on the concept of mental disorder and the biopsychosocial model are great works of practical philosophy. We managed to catch up in the midst of the COVID-19 crisis and thankfully the technology didn't let us down!

**Professor Bolton, you wrote your book on the concept of mental disorder^1^ in 2008. What is your view of the development of the knowledge in that field?**

When I wrote the book, it was prepared over several years of the MSC course that you attended and which started in the 1990s. At that time, the main contender in the philosophical and conceptual field about the concept of mental disorder was Jerome Wakefield's ‘harmful dysfunction’ account.^2^ He proposed a naturalistic view according to which, apart from any value that concerned human goods and harm, the concept of mental disorder also presupposed a natural fact, a dysfunction, which in his version was elucidated in evolutionary theoretical terms. Wakefield's account was a response to the challenges of 1960s and 1970s, which questioned whether mental disorders were ‘natural kinds’ as opposed to social constructs. My main task was to interrogate the assumptions of Wakefield's account. As it turned out, I was unable to make it work and came to the conclusion that mental disorder could not be pinned down by ‘natural facts’ as opposed to ‘social facts’ and the best way to capture it was in terms of impairment and distress, as in the DSM and ICD, which are as much personal and social as they are natural. What has changed since then (and I do not claim it is because of my book, which was at best an expression of shifting sands) is that attempts to disentangle natural facts about mental disorders have become less attractive, and what remains, the personal and social involvement of mental disorder – what might be called the psychological and social phenomenology – is more accepted. This leaves us with both challenges and opportunities.

**What are these challenges and opportunities?**

For opportunity, I think the person and the social context emerge as more central. The focus shifts from a disordered brain, or a disordered set of beliefs/behaviour, to the person in a social context with impairment/distress. The social context includes that it is not only (or not even) the patient who is distressed, but the family and extended society. The challenge is that the boundaries between illness and health, between what is and what is not the proper domain of healthcare, are more blurred, more socio-politically contentious. This of course has been recognised as an issue for psychiatry, but increasingly now in discourse about risks to physical health, evident for example in controversies over how to best manage the current pandemic.

**Speaking of the boundaries between illness and health, that reminds me of a fairly recent book called *Vagueness in Psychiatry*.**^3^ **It argues that there is an inherent vagueness in terms such as health and disorder, which are semantic properties that cannot be corrected by gathering more facts and there always will be borderline cases. The existence of vagueness would not devalue the concept on its own. Do you agree?**

It is true. I was brought up in the later Wittgenstein's philosophy school and my PhD was on works of Wittgenstein. In Wittgenstein's early work (the *Tractatus Logico-Philosophicus*) – like in the philosophy of logician and philosopher Gottlob Frege – it was assumed that concepts of course had clear boundaries. In what are called his later works (*Philosophical Investigations* is the major one), Wittgenstein dismantled the idea of clear boundaries. He likened language to a toolbox with lots of different tools for different purposes. If you look at language like that you lose the idea that concepts must have or ought to have sharp boundaries.

**With reference to definitions, in some schools of philosophy definition is identifying necessary and sufficient conditions. Could this be part of the problem, where we try to find necessary and sufficient conditions for mental disorder – could moving towards a descriptive way be helpful?**

It is an interesting question. If we think of philosophy as a canon and choose the great philosophers of the past few hundred years we see that the use of necessary and sufficient conditions does not feature or hardly features in their work. Philosophers such as Wittgenstein, Heidegger and Russell are example of this. Generally, I don't think concepts are usefully explained in terms of necessary and sufficient conditions. So, in the present context, when Robert Spitzer worked on the concept of mental disorder for DSM-III he was apparently not trying to identify necessary and sufficient conditions, but to identify criteria for its use (I believe he may have used the term ‘conceptualisation’ rather than ‘definition’).^4^ When we talk of definition of mental disorder, I think we are typically trying to conceptualise it and identify how it relates to other areas of interest, including personal distress and social impairments, as well as the various life and human sciences. Looked at like this, it is clear that mental disorder is not a fixed thing: the conceptual geography changes with changes in science and culture.

**The discussion of social context brings me to your work on the biopsychosocial model. You recently co-authored a book with Grant Gillett titled *The Biopsychosocial Model of Health and Disease*.**^5^ **What made you interested in it?**

In 2010, there was a book published with the title *The Rise and Fall of Biopsychosocial Model* by Nassir Ghaemi, whom I know.^6^ He argued that the biopsychosocial model was empty and without much use. That did puzzle me, as most people I know and whose work I had read seemed to suppose that the biopsychosocial model meant something and was the correct model for science and for practice. Nassir's book was a challenge to what the biopsychosocial model actually meant. He also added that it was a cover for loose thinking.

**I remember attending a lecture of his where he argued that the biopsychosocial model was eclectic. If I remember correctly, it followed that the model was to some degree vacuous.**

The historical context given in Nassir's book focused on the model mainly in the USA. He saw it as a framework within which competing models of psychoanalysis, social psychiatry and biological psychiatry could coexist and all be true.

**In your book with Grant Gillett,**^5^ **you explored causation at different levels. I was interested in how you separated causation at the biological level from causation at the level of physics and chemistry.**

The idea was worked out while writing the book. In his original paper, Engel identified reductionism as a problem for biomedicine, specifically reducing biology to physics and chemistry.^7^ That would exclude a distinctive biological causation and especially would exclude psychological and social causes. The core idea of the book was that there was a causation at the biological level that was above physics and chemistry. For the past 50 years or so biomedicine has developed as an exquisite combination of physics and chemistry but plus a whole new science of regulatory control, bringing in concepts such as functions, ends, positive and negative feedback systems and information flow. Importantly for the biopsychosocial model, these same causal explanatory frameworks also apply in the psychological and social domains. This is a way out of reductionism that can make sense of biopsychosocial causal interactions and the accumulating evidence of them in epidemiology and clinical therapeutics.

**I was interested that you found the core of psychological causation in agency. How did you come up with that?**

We didn't think about it at first. The primary task was to clarify causation and theoretical concepts of the various associated systems at the biological level. Once it became clearer as above, the psychological had to be understood as a system for regulating (causing) behaviour in the ‘outside world’. ‘Agency’ is a shorthand for this. Also, various pieces of the contemporary scientific jigsaw puzzle fell into place, such as that neuroscience and psychology merge into one another, and that cognition is embodied. As to health problems and biopsychosocial medicine, this approach highlights emerging findings that many physical health problems, and especially the extent of experienced pain and associated impairment, key drivers of service use in long-term conditions, involve psychosocial as well as biological factors, implicating central involvement as well as biological systems below the neck.

**In the book,**^5^ **you mentioned that we do not need to have an explicit theory of causation to accept causes in psychological and social levels. What do you think of psychiatry's engagement with the philosophy of causation?**

All medicine purports to make a difference in the lives of patients and in this sense at least it needs some causal assumptions. It is true that we do not know how some treatments work, but when we intervene in different ways, we are supposing that it makes a difference and that is why we do it. The causal assumption is essential in these kinds of applied sciences. It is not unusual to not know how a treatment works but we should not lose confidence if we have evidence from, for example, randomised controlled trials that it is effective.

**That reminds me of an anecdote on vaccination. It took several decades to know how the smallpox vaccine worked. If we wanted to wait to know the exact mechanism, millions would have died.**

True. And if we contrast it with the present state that different vaccines are proposed for COVID-19, based on detailed models of cellular mechanisms of disease progression and how to interfere with them, it shows how much this field has progressed.

**Going back to the reductionism, I found it interesting that you used emergentism as a way to challenge reductionism.**

That was interesting. I didn't try to defend emergentism, which is a slippery idea with a complex history. I understood the position simply in terms of evolution, in which increasingly complex forms of life appear, each with characteristic phenotypic traits and associated causal powers.

**I think your formulation of the biopsychosocial model is very useful. It could be very informative with concepts such as trauma.**

Trauma is of course an important and interesting topic, with a long history in psychiatry and psychology. In terms of the biopsychosocial model we propose, trauma is an environmental stressor that has a direct negative impact on agency. In defining trauma in the context of PTSD, DSM and ICD regard ‘helplessness’ as a key feature. The position is that the most salient and important outcome in the situation (the person's own survival) is out of their control. This occurs in acute situations like trauma but also in chronic exposure to severe stressors, implicated in upregulation of psychobiological stress mechanisms and raising risk of many kinds of both mental and physical health problems.

**Thank you very much for your time.**
